# Ecological, genetic and evolutionary drivers of regional genetic differentiation in *Arabidopsis thaliana*

**DOI:** 10.1186/s12862-020-01635-2

**Published:** 2020-06-22

**Authors:** Antonio R. Castilla, Belén Méndez-Vigo, Arnald Marcer, Joaquín Martínez-Minaya, David Conesa, F. Xavier Picó, Carlos Alonso-Blanco

**Affiliations:** 1 Centre for Applied Ecology “Prof. Baeta Neves”, InBIO, School of Agriculture, University of Lisbon, Lisbon, Portugal; 2 Departamento de Ecología Integrativa, Estación Biológica de Doñana (EBD), Consejo Superior de Investigaciones Científicas (CSIC), Sevilla, Spain; 3 Departamento de Genética Molecular de Plantas, Centro Nacional de Biotecnología (CNB), Consejo Superior de Investigaciones Científicas (CSIC), Madrid, Spain; 4 CREAF, Centre de Recerca Ecològica i Aplicacions Forestals, Bellaterra, E08193 Cerdanyola de Vallès, Catalonia Spain; 5 Universitat Autònoma de Barcelona, Bellaterra, E08193 Cerdanyola de Vallès, Catalonia Spain; 6 BCAM - Basque Center for Applied Mathematics, Bilbao, Spain; 7 Departament d’Estadística i Investigació Operativa, Universitat de València, Valencia, Spain

**Keywords:** Genetic diversity, Genetic structure, Iberian Peninsula, Nested maximum-likelihood population effect models, Precipitation seasonality, Spatial hierarchical Bayesian models

## Abstract

**Background:**

Disentangling the drivers of genetic differentiation is one of the cornerstones in evolution. This is because genetic diversity, and the way in which it is partitioned within and among populations across space, is an important asset for the ability of populations to adapt and persist in changing environments. We tested three major hypotheses accounting for genetic differentiation—isolation-by-distance (IBD), isolation-by-environment (IBE) and isolation-by-resistance (IBR)—in the annual plant *Arabidopsis thaliana* across the Iberian Peninsula, the region with the largest genomic diversity. To that end, we sampled, genotyped with genome-wide SNPs, and analyzed 1772 individuals from 278 populations distributed across the Iberian Peninsula.

**Results:**

IBD, and to a lesser extent IBE, were the most important drivers of genetic differentiation in *A. thaliana*. In other words, dispersal limitation, genetic drift, and to a lesser extent local adaptation to environmental gradients, accounted for the within- and among-population distribution of genetic diversity. Analyses applied to the four Iberian genetic clusters, which represent the joint outcome of the long demographic and adaptive history of the species in the region, showed similar results except for one cluster, in which IBR (a function of landscape heterogeneity) was the most important driver of genetic differentiation. Using spatial hierarchical Bayesian models, we found that precipitation seasonality and topsoil pH chiefly accounted for the geographic distribution of genetic diversity in Iberian *A. thaliana*.

**Conclusions:**

Overall, the interplay between the influence of precipitation seasonality on genetic diversity and the effect of restricted dispersal and genetic drift on genetic differentiation emerges as the major forces underlying the evolutionary trajectory of Iberian *A. thaliana*.

## Background

Genetic diversity is an important asset for the ability of populations to adapt and persist in changing environments [1–8]. The spatio-temporal changes in genetic diversity constantly taking place in any population—regardless of the causes, pace and the phenotypic effects of such changes—constitute the raw material upon which natural selection eventually acts [9–11]. At any spatial scale, genetic diversity typically becomes unevenly distributed across space [12], as genetic diversity is determined by how genetic diversity is partitioned within and among populations across the distribution. In other words, the spatial distribution of genetic diversity depends on the extent of genetic differentiation among populations whatever the sources of such differentiation. Nonetheless, it must be noted that genetic differentiation is a spatially-explicit phenomenon, as genetic differentiation strictly depends on the genetic relationship that a given population has with its neighbors near and far.

The inherent spatial nature of genetic differentiation defines the theoretical and methodological framework of three models, which are not mutually exclusive, testing the major drivers of genetic differentiation: isolation-by-distance (IBD hereafter), isolation-by-environment (IBE hereafter) and isolation-by-resistance (IBR hereafter) models. In the classical IBD, genetic differentiation among populations exhibits a positive relationship with geographic distance [13–18]. In this case, dispersal limitation and genetic drift determine the greater genetic differentiation at larger distances. In fact, limited dispersal constrains gene flow among populations, which is not able to counteract the effect of genetic drift within populations [16–18]. Many types of organisms exhibit IBD [19], probably because unrestricted gene flow hardly occurs in nature. Nevertheless, quantifying the contribution of limited dispersal and genetic drift to genetic differentiation is not a straightforward task, as we largely ignore the actual extent of dispersal, the effective population sizes conditioning genetic drift, and the effects of historical factors shaping the genetic relationships among populations [20].

In contrast, the IBE model deals with the effects of environmental differences on genetic differentiation. IBE posits that gene exchange is strongest among populations located in similar environments, which would be mediated by environmental heterogeneity, the extent of local adaptation and spatial variation in gene flow across space [18, 21, 22]. Thus, genetic differentiation among populations increases with their environmental differentiation, independently of their geographic distance. IBE can arise due to multiple factors, such as biased dispersal due to preferences for particular environments, natural selection against maladapted immigrants, sexual selection against immigrants when they exhibit divergence in mating choices or sexual signals, and natural selection against hybrids when they show reduced fitness relative to non-hybrids [22].

Finally, the IBR model takes environmental heterogeneity across landscapes into account as a modulator of gene flow and its effects on genetic differentiation [23]. The IBR model predicts a positive relationship between genetic differentiation and resistance distance among populations [24–26]. The resistance distance between population pairs is a concept inspired in circuit theory, which considers the landscape features reducing the probability of dispersal and gene flow among populations [24–27]. Under IBR, the spatial structure of habitat suitability is of paramount importance to determine the least cost path between population pairs optimizing their connectivity and thus minimizing their resistance distance (24 and references therein). Given that the resistance distance between population pairs is a function of geographic distance, and that presence-background models estimate habitat suitability using environmental predictors, IBR inevitably conflates IBD and IBE [22].

Here, we tested these three hypotheses to identify the major drivers of genetic differentiation in the annual plant *Arabidopsis thaliana* across the Iberian Peninsula. This is the region of the species’ distribution harboring the largest genomic diversity [28–30]. In addition, Iberian *A. thaliana* occurs in a wide array of natural environments practically across the whole region, spanning from seaside to sub-alpine locations [31–33]. These two features are likely the result of *A. thaliana*’s history in the Iberian Peninsula [29, 31, 34], where the species long survived by developing adaptations to ample environmental heterogeneity over dramatic climatic oscillations. The occurrence of relict populations with an African origin [29, 34] also supports such history of Iberian *A. thaliana*. In fact, habitat suitability of relict populations has been associated to more stable vegetation dynamics since the Last Glacial Maximum and during the Holocene in the Iberian Peninsula [35]. Overall, the geographic ubiquity, the large amount of genetic diversity, the broad variety of habitats occupied, and the long evolutionary history make Iberian *A. thaliana* an appropriate study system to disentangle the drivers of genetic differentiation at a regional scale.

Given the fact that a small sample size seriously reduces power and accuracy of spatial analyses [36], IBD, IBE and IBR were tested using 278 Iberian *A. thaliana* populations collected over a decade. About six individuals per population, totaling 1772 individuals, were genotyped with genome-wide, putatively neutral SNPs to estimate genetic diversity, differentiation and structure. We hypothesized that IBD and IBE largely accounted for genetic differentiation for two reasons. First, IBD is a common result in *A. thaliana* genetic studies—including the Iberian Peninsula [31–33]—due to dispersal limitation and high self-fertilization rates. Second, Iberian *A. thaliana* shows significant adaptive variation in fitness-related traits across environmental gradients [30, 32, 37, 38]. However, we ignore the importance of IBR and the joint contribution of IBD, IBE and IBR to the genetic differentiation of Iberian *A. thaliana* populations. For the sake of completeness, we also examined the geographical distribution of genetic diversity in Iberian *A. thaliana*. To this end, we employed a spatial hierarchical Bayesian model to identify regional hot and cold spots of genetic diversity and their potential environmental predictors. Overall, we stress the importance of identifying the drivers of genetic differentiation among populations, but also of pinpointing the forces that determine the amount of genetic diversity within populations, to understand the evolutionary dynamics of any organism.

## Results

### Genetic diversity

**Fig. 1:**
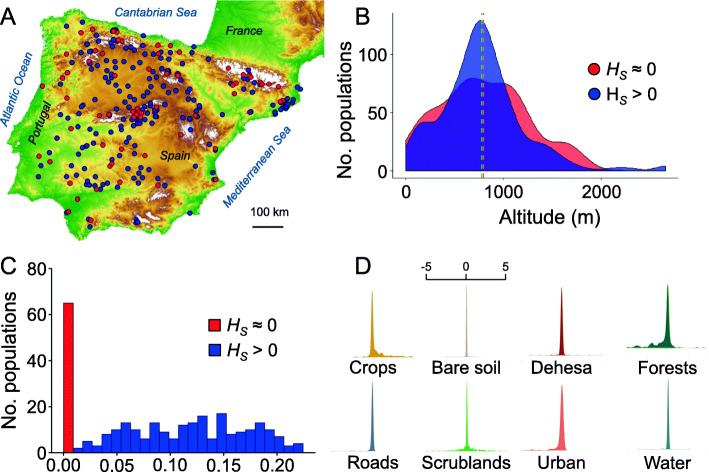
Distribution of populations, genetic diversity and temporal habitat changes of Iberian *Arabidopsis thaliana* populations. **a** Geographic distribution of the 278 *A. thaliana* populations of study across the Iberian Peninsula. Red and blue dots represent populations with zero and non-zero genetic diversity (*H*_*S*_) values, respectively. **b** Frequency distribution of populations with zero and non-zero *H*_*S*_ values as a function of altitude. Mean altitude for both groups of populations is almost identical, as indicated by dashed lines. **c** Frequency distribution of populations with zero and non-zero *H*_*S*_ values. **d** Frequency distribution of the average percentage change between year intervals for each habitat type. Data from digitalized orthophotographs available from each population. For the sake of clarity, only one X-axis is shown, indicating the accumulation of populations with average percentage changes around zero. The map of Fig. 1a was obtained from the National Center for Geographic Information (CNIG) of Spain

We genotyped 240 genome-wide SNPs in 1172 individuals collected from 278 populations (Fig. 1a) to determine the genetic diversity of *A. thaliana* in the Iberian Peninsula. In this set of populations, the percentage of polymorphic loci (PL) ranged between 0 and 56.7% (mean ± SD = 24.4 ± 17.7%), the mean number of observed alleles per locus (*n*_*a*_) between 0.95 and 1.56 (mean ± SD = 1.23 ± 0.18), and mean gene diversity (*H*_*S*_) between 0 and 0.224 (mean ± SD = 0.09 ± 0.07). *H*_*S*_ exhibited a bimodal distribution clearly differentiated in two groups of populations. On the one hand, 66 populations had very low *H*_*S*_ values (54 of 0 and 12 between 0.001 and 0.009; Fig. 1c). On the other hand, the remaining 212 populations had *H*_*S*_ values distributed between 0.015 and 0.224 (Fig. 1c). The 66 populations with no genetic diversity did not show geographic or environmental bias, as they occurred scattered across the region (Fig. 1a) and the altitude gradient (Fig. 1b). Average (± SD) genetic differentiation, given by pairwise *F*_*ST*_ values, including all populations was of 0.644 ± 0.189 (*F*_*ST*_ = 0.538 ± 0.150 without the 66 populations with no genetic diversity).

Overall, we found 1613 non-redundant multilocus genotypes (*N*_*H*_) in the 1772 individuals, which showed an average proportion of allelic differences between haplotype pairs of 0.30 ± 0.05. We only found five non-redundant multilocus genotypes in different populations. In particular, two (separated by 0.6 km) and three populations (separated by 7.6–12.8 km) included the same non-redundant multilocus genotypes.

To assess whether populations experienced major or sudden disturbances that could affect their genetic diversity and differentiation during the last decades, we retrieved temporal series of publicly available orthophotographs from all populations. The analyses of the habitats interpreted from digitalized orthophotographs indicated that land-used changes and major disturbances (wildfires, landslides or the development of large infrastructures) over the time considered did not affect landscapes. In particular, the average change per habitat type between year intervals was concentrated around zero (Fig. 1d), indicating that habitats barely changed over time. We ignore, however, whether pathogen or diseases affected *A. thaliana* populations, which could influence genetic diversity patterns. We have never observed noticeable catastrophic events caused by pests and diseases over 15-plus years of experience sampling natural *A. thaliana* populations (C. Alonso-Blanco and F. X Picó, pers. obs.).

### Geographic distribution of genetic diversity

**Fig. 2:**
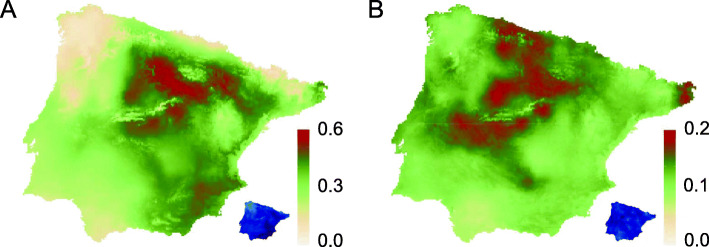
Geographic distribution of genetic diversity within Iberian *Arabidopsis thaliana* populations estimated by spatial hierarchical Bayesian modeling. **a** Distribution of populations with zero (*N* = 66; absence of genetic diversity) and non-zero (*N* = 212; presence of genetic diversity) genetic diversity (*H*_*S*_) values. Darker and lighter intensities indicate higher and lower odds for populations with non-zero and zero *H*_*S*_ values, respectively. **b** Distribution of populations with *H*_*S*_ values higher than zero (*N* = 212). Darker and lighter intensities indicate higher and lower *H*_*S*_ values, respectively. In both cases, the uncertainty of spatial hierarchical Bayesian model is given as standard deviation units in small maps. Darker and lighter intensities indicate higher and lower uncertainty, respectively

To determine the geographic and environmental distribution of *H*_*S*_ in *A. thaliana* populations, we used a spatial hierarchical Bayesian modeling to explain *H*_*S*_ as a function of environmental variables. The model considered simultaneously the populations with zero (*N* = 66 populations) and non-zero (*N* = 212 populations) *H*_*S*_ values. The best fitting model indicated that the occurrence probability of populations with non-zero *H*_*S*_ values (populations with genetic diversity) was concentrated towards the center and eastern areas of the Iberian Peninsula (Fig. 2a). In contrast, northern, western and southern peripheral areas had higher odds for populations lacking genetic diversity (Fig. 2a). In addition, genetic diversity of *A. thaliana* was unevenly distributed across the Iberian Peninsula, with different nuclei with high diversity in large central and northern areas, as well as in a limited area in NE Spain (Fig. 2b). We did not detect any anomaly in the distribution of the mean effects of the spatial component and its uncertainty (Fig. S2) that made us suspect that the spatial effects were affecting the results.

The environmental predictors with more influence on the spatial distribution of *H*_*S*_ were precipitation seasonality (BIO15), precipitation of the warmest quarter (BIO18) and topsoil pH. The contributions of precipitation variables were negative for the occurrence of populations with no genetic diversity, as well as for the genetic diversity of populations with non-zero *H*_*S*_ values (Table 1). Overall, these results indicated that areas with higher precipitation seasonality, and to a lesser extent higher precipitation in the warmest quarter, tended to harbor populations with lower genetic diversity. In the Iberian Peninsula, higher and lower precipitation seasonality characterizes xeric and mesic environments, respectively. The contribution of topsoil pH was positive for the occurrence probability of populations with zero or non-zero *H*_*S*_ values, meaning that populations with non-zero *H*_*S*_ values tended to occur in areas with basic soils. However, in the basic areas of the Iberian Peninsula (E and SE Spain) the species is very rare (Fig. 1 and S1), and the low number of populations there with non-zero *H*_*S*_ values could be introducing some bias in the model. In contrast, the contribution of topsoil pH was negative for genetic diversity, indicating that populations with higher genetic diversity tended to occur in acidic soils, which is consistent with the preference of the species for this sort of soils (C. Alonso-Blanco and F.X. Picó, pers. obs.).

**Table 1:** Coefficients of the best spatial hierarchical Bayesian model for the geographic distribution of genetic diversity of Iberian *Arabidopsis thaliana*

	*H*_*S*_ ≈ 0		*H*_*S*_ > 0	
Variables	*β*	SD	*β*	SD
Intercept	2.054	1.776	0.305	0.604
BIO15	−0.025	0.017	−0.023	0.006
BIO18	−0.009	0.004	−0.004	0.001
Topsoil pH	0.176	0.200	−0.187	0.067

Entries (*β* ± SD) are given for the degenerate distribution with point mass and zero (binary data including populations with *H*_*S*_ values ≈ 0) and a conditional-to-presence continuous process (continuous data including populations with non-zero *H*_*S*_ values). The best model had a WAIC value of − 365.43 and a LCPO value of − 0.31. BIO15 is the precipitation seasonality and BIO18 is the precipitation of the warmest quarter

### Genetic structure

**Fig. 3:**
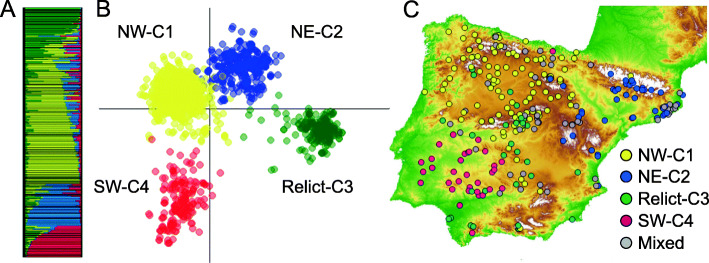
Genetic structure of the 278 *Arabidopsis thaliana* populations of study across the Iberian Peninsula depicting the four genetic clusters (NW-C1, NE-C2, relict-C3 and SW-C4). **a** Results from the Bayesian clustering method implemented in STRUCTURE. **b** Results from the Discriminant Analysis of Principal Components (DAPC). **c** Geographic distribution of homogeneous populations from each genetic cluster. Homogeneous populations (*N* = 230) were those with average membership proportions among individuals within populations greater than 0.3 for only one genetic cluster. Mixed or heterogeneous populations (*N* = 48) are also shown in grey. The map of Fig. 3c was obtained from the National Center for Geographic Information (CNIG) of Spain

We analyzed the structure of the 278 *A. thaliana* populations in different genetic clusters with the 1613 non-redundant multilocus genotypes using Bayesian (Fig. 3a) and ordination (Fig. 3b) methods. Both analyses detected four genetic clusters, three of them showing a strong geographic structure (Fig. 3c): the northwestern cluster 1 (NW-C1 hereafter), the northeastern cluster 2 (NE-C2 hereafter), and the southwestern cluster 4 (SW-C4 hereafter; Fig. 3b). In contrast, cluster 3, which corresponded to the group previously described as the relict *A. thaliana* lineage (relict-C3 hereafter), occurred scattered across the Iberian Peninsula (Fig. 3c). These results were fully consistent with previous studies on Iberian *A. thaliana* based on just one accession per population [31, 32, 34, 39].

Since we used about six individuals per population, we could classify populations as homogeneous or heterogeneous based on the assignment of individuals to a single or multiple genetic clusters, respectively. Most populations (230 of 278) were homogeneous, with 118, 44, 35 and 33 belonging to genetic clusters NW-C1, NE-C2, relict-C3 and SW-C4, respectively (Fig. 3c). The remaining 48 heterogeneous populations were distributed across the region, with an accumulation of them in central and NE Spain (Fig. 3c). As expected, the most abundant genetic clusters, NW-C1 and NE-C2, exhibited a higher number of heterogeneous populations, because 46 of 48 heterogeneous populations were included in these genetic clusters.

### Drivers of genetic differentiation

**Fig. 4:**
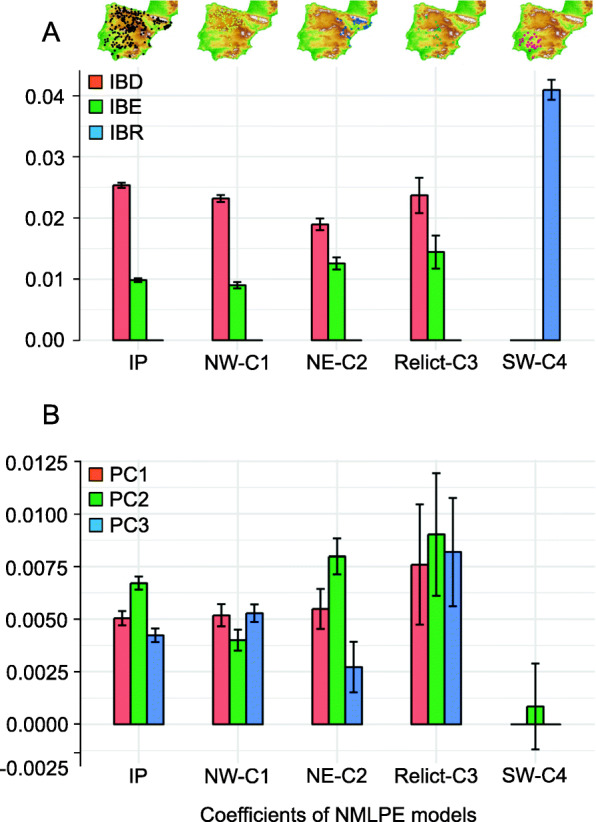
Effects of isolation-by-distance (IBD), isolation-by-environment (IBE) and isolation-by-resistance (IBR) on genetic differentiation in *Arabidopsis thaliana*. **a** Coefficients (± SD) of the top-ranked nested maximum-likelihood population effects models (NMLPE) testing the effect of IBD, IBE and IBR on genetic differentiation in *A. thaliana*. **b** Model averaged coefficients (± SD) for three Principal Component (PC) axes accounting for more than 75% of the total variance. Model averaging was conducted using the subsample of models exhibiting ∆AIC < 2 regarding the top-ranked model, if more than one. In all cases, model estimates for the analysis conducted for the entire Iberian Peninsula (IP) and the four genetic clusters (NW-C1, NE-C2, relict-C3, and SW-C4) are shown. Maps with the geographic distribution of populations used in each analysis are also given. Maps were obtained from the National Center for Geographic Information (CNIG) of Spain

We applied nested maximum-likelihood population effect models (NMLPE) to evaluate simultaneously the effects of the geographic (IBD), environmental (IBE) and resistance (IBR) variation on the genetic differentiation among Iberian *A. thaliana* populations. IBD and IBE accounted for genetic differentiation at the entire Iberian Peninsula scale (Table 2A and Fig. 4a). In addition, we dissected IBE into three environmental PCs, which substantially contributed to the IBE (Table 2A and Fig. 4b). These PCs explained 29.3, 23.8, and 22.2% of the environmental variance, respectively, which accounted for different environmental gradients across the Iberian Peninsula. In particular, PC1 represented a gradient of temperature and precipitation, pinpointing the negative relationship between the two variables in the Iberian Peninsula. PC2 only depicted the gradient of temperature across the region. Finally, PC3 illustrated a gradient of precipitation and pH in which soils with lower pH receive higher precipitation in the Iberian Peninsula.

**Table 2:** Summary statistics for the top-ranked NMLPE models evaluating the effect of isolation-by-distance (IBD), isolation-by-environment (IBE) and isolation-by-resistance (IBR) on the genetic differentiation among Iberian *Arabidopsis thaliana* populations

Cluster	Predictors	AIC	∆AIC	Weight
A – Model: IBD + IBE + IBR				
IP	IBD + IBE	−128,716.6	0.00	1.00
NW-C1	IBD + IBE	−42,836.0	0.00	1.00
NE-C2	IBD + IBE	−10,406.3	0.00	1.00
Relict-C3	IBD + IBE	− 2938.5	0.00	1.00
SW-C4	IBR	− 3753.4	0.00	0.79
B – Model: IBD + PC1 + PC2 + PC3 + IBR				
IP	IBD + PC1 + PC2 + PC3	−128,694.0	0.00	1.00
NW-C1	IBD + PC1 + PC2 + PC3	−42,856.2	0.00	1.00
NE-C2	IBD + PC1 + PC2 + PC3	−10,380.3	0.00	0.83
Relict-C3	IBD + PC1 + PC2 + PC3	− 2936.6	0.00	0.89
SW-C4	IBR	−3753.4	0.00	0.41
	IBR + PC2	− 3751.6	1.84	0.17

Entries are given for the entire Iberian Peninsula (IP) and the four genetic clusters detected using all populations available (*N* = 278). The two models include IBD, IBE and IBR, and IBE given the principal analysis components (PCs). Akaike information criterion (AIC), ∆AIC and model weight are reported

We found that the single model including IBD and IBE was ranked as the top model for three genetic clusters (NW-C1, NE-C2, and relict-C3) with no models differing less than 2 in AIC (Table 2B and Fig. 4a). Although both IBD and IBE had significant effects on genetic differentiation, size effects were greater for IBD than for IBE (Fig. 4a). In addition, the three PC axes made substantial contributions to the IBE exhibited by the populations for the same three genetic clusters (Table 2B and Fig. 4b). For SW-C4, a single model including IBR was ranked as the top model (Table 2B and Fig. 4a). When environmental PCs were included separately in the analyses, we found two top-ranked models exhibiting ∆AIC < 2 (Table 2) for SW-C4. However, model averaging discarded a relevant role of PC2 on the genetic differentiation among SW-C4 populations (Fig. 4b).

## Discussion

Genetic differentiation is a dynamic process because any population is constantly under the effect of ecological, genetic and evolutionary forces modifying the amount of genetic diversity within and among populations. Disentangling such forces accounting for genetic differentiation, which underlies major evolutionary processes from local adaptation to speciation, has long promoted the development of a strong theoretical and methodological framework since practically the birth of the Modern Synthesis. Here, we addressed this question by testing three hypotheses (IBD, IBE and IBR) accounting for regional-scale genetic differentiation in Iberian *A. thaliana*.

The main result of our study, based on nested maximum-likelihood population effect models (NMLPE), indicated that genetic differentiation in *A. thaliana* was mostly accounted for by IBD, and to a lesser extent by IBE (Fig. 4a). In other words, dispersal limitation, genetic drift, and to a lesser extent local adaptation, determined the distribution of *A. thaliana*’s genetic diversity within and among populations across its Iberian range. This result extends previous studies showing a marked IBD in *A. thaliana* in the Iberian Peninsula [30–33] and elsewhere [28, 34, 40–48]. It is widely accepted that IBD in *A. thaliana* results from the joint effects of dispersal limitation, self-fertilization, local adaptation and demographic history. Our study contributed to disentangle the effect of some of these factors, suggesting that dispersal limitation and genetic drift are probably more efficient than local adaptation in shaping genetic differentiation patterns in *A. thaliana*. We found additional support to this conclusion in the low number of populations located at short distances sharing the same multilocus genotypes, stressing the limited natural *A. thaliana*’s dispersal ability [41, 47–49].

In contrast, IBR did not account for genetic differentiation at a regional scale (Fig. 4a), suggesting that landscape heterogeneity was not relevant for genetic differentiation in Iberian *A. thaliana*. We believe that the lack of IBR found across the Iberian Peninsula is likely due to the species’ cosmopolitan habit and a distribution without major discontinuities (Fig. S1). Thus, most population pairs are connected by relatively high habitat suitability, diminishing resistance distances. The demographic history, characterized by the presence of the relict African lineage and other non-relict Eurasian lineages [28–31, 34, 35, 50], and the well-known ability to adjust flowering time and seed dormancy to contrasting environments [38, 51–55], both account for the cosmopolitan habit of Iberian *A. thaliana*.

We also explored the effect of IBD, IBE and IBR for each of the four genetic clusters, which represent the joint outcome of the long demographic and adaptive history of the species in the region [30–32, 34, 39, 56]. Overall, NW-C1, NE-C2 and relict-C3 exhibited the same patterns than those at the regional scale, i.e. IBD and IBE mostly accounted for genetic differentiation within each cluster (Fig. 4a). However, it is worth noting that the importance of IBE was not the same for these three clusters, since NE-C2 and relict-C3 exhibited higher IBE than NW-C1 (Fig. 4a), stressing probably the higher influence of local adaptation for genetic differentiation in these clusters. In fact, NE-C2 is a cluster with pronounced altitudinal gradients in NE Spain (from maritime to sub-alpine environments) along which the species is known to have adapted by adjusting various life-cycle and physiological traits [57–61]. In the case of relict-C3, the long evolutionary history of this lineage has provided the means to survive and adapt to diverse Iberian environments over the last millennia [35]. On the contrary, that was not the case for SW-C4, whose genetic differentiation was accounted for by IBR (Fig. 4a). Whereas SW-C4 is the genetic cluster with the most restricted geographic distribution (Fig. 3c), its populations are located across an area showing dramatic changes in habitat suitability (Fig. S1). Most likely, this particularity accounted for the enormous weight of IBR in this cluster.

Finally, we also conducted NMLPE using principal component (PC) axes to pinpoint the major environmental predictors underlying IBE. We found that the three PCs depicting gradients of temperature and/or precipitation across the region made substantial contributions to IBE in NW-C1, NE-C2 and relict-C3 Iberian clusters (Fig. 4b), probably due to the similar contributions of the three PCs to the environmental variation among these populations. In addition, as IBE informs on the influence of local adaptation on genetic differentiation, our results reinforce the view of the strong effects of environmental gradients on local adaptation in Iberian *A. thaliana*. For example, previous studies showed that geographic variation in fitness-related traits in Iberian *A. thaliana* (flowering time and seed dormancy) strongly co-varied as a function of temperature and to a lesser extent of precipitation [30, 32, 33, 38, 55, 62, 63]. In particular, *A. thaliana* adjusts its life cycle by advancing flowering time and increasing seed dormancy as the environment becomes warmer, drier and more seasonal [38, 55, 63].

As genetic diversity and its within- and among-population distribution patterns represents the raw material upon which genetic differentiation is estimated, we discuss some results dealing with genetic diversity of Iberian *A. thaliana* that are worth considering. For example, analyses of the genetic diversity in this large number of populations showed that 24% of them had no or very low genetic diversity (Fig. 1a–c). This is not exceptional as other authors also detected populations with practically no genetic diversity in *A. thaliana* [40, 41, 49, 64, 65]. Strong founder effects [66] and low migration rates [41] could well account for this result. Furthermore, small *A. thaliana* populations rather isolated and locally adapted to particular environments can have very low genetic diversity [67], a scenario that could also apply to some of our populations. Finally, sampling bias might also affect genetic diversity [68], particularly because some populations were sampled late in the reproductive season.

Whatever the probable combination of factors accounting for the existence of *A. thaliana* populations with no or very low genetic diversity, the spatial hierarchical Bayesian model allowed the analysis of the geographic distribution of genetic diversity in Iberian *A. thaliana* (Fig. 2), particularly to detect the environmental predictors of the geographic distribution of genetic diversity. Precipitation, and not temperature, emerged as the major environmental factor accounting for the split of populations with and without genetic diversity, as well as for genetic diversity (Table 1). This result is in agreement with other studies on the distribution of genetic diversity in plants, which indicated a trend for a higher role of precipitation variables over those of temperature [69–74]. In addition, precipitation seasonality, which determines climatic variation in the Iberian Peninsula spanning from drier Mediterranean to more humid Atlantic climates, was the most important bioclimatic variable for the geographic distribution of genetic diversity in *A. thaliana* (Table 1). Previous studies also found that precipitation seasonality was a good predictor of individual fitness [33] and distribution of the four genetic clusters [39]. Albeit precipitation seasonality may be hard to handle as a fixed factor, further experiments are needed to better understand how *A. thaliana* responds to this variable, which might be a major evolutionary force in this species.

## Conclusions

This study dissected the complexity of geographic, environmental and evolutionary factors contributing to the genetic differentiation among *A. thaliana* populations, while illustrating the power of dense collections to disentangle complex biological questions [8, 28, 30, 49, 63, 75, 76]. Beyond genetic differentiation patterns, we also identified the environmental predictors accounting for genetic diversity within populations. Clearly, the processes driving among-population genetic differentiation may not be the same than those determining the amount of within-population genetic diversity. However, we need to deal with the latter to understand the former. In this sense, recent resurrection studies showed that natural *A. thaliana* populations exhibited substantial changes in their genetic composition in just a decade [77, 78]. The extent of temporal genetic variation in *A. thaliana* represents a reminder that populations are not static, and that the geographic distribution of genetic diversity within and among-populations is changing too. Repeated genomic scans over time on the same populations will provide real-time insights into the intensity and pace of genetic variation within populations, which will increase our understanding of the evolutionary processes shaping genetic differentiation in plant populations.

## Methods

### Source populations

We sampled 278 natural populations of the annual plant *Arabidopsis thaliana* (L.) Heyhn. (Brassicaceae) across the entire Iberian Peninsula (~ 800 × 700 km^2^; 36.00–43.48°N, 3.19ºE–9.30°W; Fig. 1a) during the decade of the 2000s. C. Alonso-Blanco and F.X. Picó identified and collected all the material. No voucher specimens of this material were deposited in a publicly available herbarium. For each population, we recorded its geographic coordinates and altitude using a GPS (Garmin International, Inc., Olathe, KS, USA). We extracted environmental data from publicly available repositories, such as WorldClim v.2 [79]; https://www.worldclim.org/; accessed 6 June 2019), The CORINE Land Cover 2000 (https://land.copernicus.eu/pan-european/corine-land-cover; accessed 6 June 2019), and The Soil Geographical Database from Eurasia v.4 (https://esdac.jrc.ec.europa.eu/tags/soil-geographical-database-eurasia; accessed 6 June 2019).

Geographic distance among populations varied between 1 and 1059 km (Fig. 1a) and altitude between 1 and 2662 m.a.s.l. (Fig. 1b). For this set of populations, annual mean temperature varied between 5.3 and 18.4 °C (mean ± SD = 12.3 ± 2.7 °C), annual total precipitation between 216.2 and 1778.8 mm (mean ± SD = 760.9 ± 289.5 mm), percentage of natural vegetation (i.e. forests, scrublands or grasslands) between 0 and 100% (mean ± SD = 61.3 ± 35.7%), and soil pH between 3.6 and 7.5 (mean ± SD = 5.7 ± 0.8).

Natural *A. thaliana* populations are made of patches of individuals widely differing in size and density, which is important when it comes to design a sampling scheme to study the spatio-temporal distribution of genetic diversity in *A. thaliana* [49, 57, 65, 77]. For each population and whenever possible, we collected seeds from several individuals from different patches (separated 1–20 m from each other) representing well each study population. Every sampling year and a few months after sampling, we multiplied field-collected seeds by the single seed descent method, in a glasshouse at the Centro Nacional de Biotecnología (CNB-CSIC) in Madrid. Bulked seeds were stored in dry, dark conditions in cellophane bags at room temperature, storing conditions that can preserve *A. thaliana* seeds for years.

In this study, each population included about six individuals, ranging between four and seven. Given the importance to work with a dense collection of natural populations, we selected this number as a compromise between the number of populations and the number of individuals per population that could be handled to extract the genetic patterns of interest. This choice was also based on a previous large-scale study exploring the within- and among-population partitioning of genetic diversity in *A. thaliana*, which yielded interpretable results with four individuals per population [40]. Besides, other studies using more individuals per population and different markers, albeit at smaller geographical scales, also found populations with no or very low genetic diversity (see below), indicating that is not unusual in *A. thaliana* [40, 41, 49, 64, 65, 77].

We collected all seeds from wild populations. *Arabidopsis thaliana* is a common plant species not categorized as protected or endangered in any species list of the Convention on the Trade in Endangered Species of Wild Fauna and Flora. We carried out field sampling in locations where no permission was required, except at Doñana National Park (permission issued by Estación Biológica de Doñana) and Sierra de Grazalema Natural Park (permission issued by Red Andaluza de Jardines Botánicos de la Consejería de Medio Ambiente y Ordenación del Territorio de la Junta de Andalucía). Based on the Royal Decree of the Spanish legislation (Real Decreto 124/2017, de 24 de febrero; https://www.boe.es/eli/es/rd/2017/02/24/124), the genetic resources included in this study fall within the definition of “exclusively for taxonomic purposes” as they were used for scientific, educational or non-commercial purposes.

### Historical factors

Recent historical changes in land use or large-scale perturbations may dramatically affect the amount of genetic diversity in plant populations. Such changes can be a misleading factor when attempting to evaluate the joint effects of IBD, IBE and IBR on genetic differentiation. This is because IBD, IBE and IBR do not contemplate perturbations producing sudden or erratic changes in genetic diversity that eventually may mask the patterns of interest. To make sure that historical factors were not an issue in this study, we estimated the temporal landscape dynamics of all study populations. We examined all aerial orthophotographs available for each population (spanning between 1945 and 2016), which were retrieved from different public regional administrations in Spain [77] with a tool specifically developed for this purpose. We excluded up to 15 populations because aerial orthophotographs were not available. The final number of aerial orthophotographs was highly variable per population, ranging between 4 and 24 (mean ± SE = 8.6 ± 0.3 aerial orthophotographs) and covering between 9 and 71 years (37.1 ± 1.7 years between the first and the last orthophotograph).

To quantify temporal landscape dynamics, a circular area (500 m radius) around the GPS coordinate was divided into a regular grid of 80 squares (100 m side) for each aerial orthophotograph available per population. Based on vegetation and land use, each square was categorized as forests, scrublands, dehesa (i.e. agro-silvicultural ecosystems based on Mediterranean oak woodlands), bare soil, crops, urban, infrastructures, and water, when one of these categories occupied at least more than half of the square. Categories were assigned to each square by digitalizing manually all squares from all aerial orthophotographs available (*N* = 2252 orthophotographs) with QGIS v.3.4 [80]. For each habitat type and population, we estimated the average change between year intervals available for each population to quantify landscape changes over time.

### SNP genotyping

A total of 1772 *A. thaliana* individuals from 278 populations were genotyped with 245 presumably neutral nuclear SNPs using the SNPlex technique (Applied Biosystems, Foster City, CA, USA) through the CEGEN Genotyping Service (www.usc.es/cegen/). These genome-wide SNPs are frequent polymorphisms in Central Europe, the Iberian Peninsula, and worldwide collections, which altogether minimized ascertainment bias [31, 32, 34]. On average, there were about 49 SNPs per chromosome (range = 45–53 SNPs) located at approximately 0.5 Mb from each other (range = 0.11 Kb – 1.82 Mb). Four SNPs had percentages of missing data above 25% and one was monomorphic. We discarded these five SNPs from the analyses maintaining 240 SNPs.

### Genetic analyses

For each *A. thaliana* population, we calculated the percentage of polymorphic loci (PL), the mean number of observed alleles per locus (*n*_*a*_) and mean gene diversity (*H*_*S*_) using FSTAT v.2.9.3 [81]. In addition, we computed the percentage of differences among all pairs of non-redundant multilocus genotypes. We used the ‘pairwise.WCfst’ function implemented in the R package hierfstat v.0.04–22 [82] to estimate genetic differentiation as pairwise *F*_*ST*_ values according to Weir and Cockerham [68], which represents the dependent variable to test IBD, IBE and IBR (see below).

The genetic structure of *A. thaliana* populations across the Iberian Peninsula was examined using two different methods: the Bayesian clustering method implemented in STRUCTURE v.2.3.3 [83, 84] and the ordination method represented by the Discriminant Analysis of Principal Components (DAPC) [85] available in the R package adegenet v.2.1.1 [85]. In the case of STRUCTURE, model settings included haploid non-redundant multilocus genotypes, correlated allele frequencies between populations and a linkage model. We identified the number of identical multilocus genotypes using the ‘mlg.filter’ function implemented in adegenet. Each run consisted of 50,000 burn-in MCMC iterations and 100,000 MCMC after-burning repetitions for parameter estimation. To determine the K number of ancestral populations and the ancestry membership proportions of each accession in each population, we ran the algorithm 20 times for each defined number of groups (K value) from 1 to 10. The number of distinct genetic clusters was determined by evaluating the differences between the data likelihood for successive K values. The largest K value with significantly higher likelihood than that of K-1 runs (two-sided *P* < 0.005; Wilcoxon tests for related samples) gave the final K number. This was supported by a high similarity among the ancestry membership matrices from different runs of the same K value (H′ = 0.99). We used CLUMPP v.1 [86] to calculate the average symmetric similarity coefficient H′ among runs and the average matrix of ancestry membership proportions, derived from the 10 runs having the highest likelihood. STRUCTURE simulations were conducted at the The Supercomputing Center of Galicia (CESGA; http://www.cesga.es/).

For the DAPC analysis, we assessed the number of clusters using the ‘find.cluster’ function in adegenet, which runs successive K-means clustering with an increasing number of clusters to determine the best supported number of genetic clusters using the Bayesian Information Criterion (BIC). The K value with the lowest BIC value represents the optimal number of clusters, although BIC values may keep decreasing after the true K value in case of genetic clines and hierarchical structure [87]. Therefore, the rate of decrease in BIC values was visually examined to identify values of K, after which BIC values only decreased in a subtle manner [87]. The ‘dapc’ function was used with the final K value, retaining the axes of the Principal Component Analysis.

### Geographic distribution of genetic diversity

We modeled the effects of environmental heterogeneity on the spatial distribution of genetic diversity by using a modified version of a spatial hierarchical Bayesian model recently used to model the effects of warming on the Iberian *A. thaliana*’s distribution [56]. The aim of this model was to visualize hot and cold spots of genetic diversity across the Iberian Peninsula and to identify their environmental predictors.

We used a Bayesian approach to handle the particularities of genetic diversity data, such as the semi-continuous nature of the variable. In the case of Iberian *A. thaliana*, 66 of 278 populations had very low mean gene diversity (*H*_*S*_) values (*H*_*S*_ < 0.009), whereas the rest of populations exhibited non-zero *H*_*S*_ values (Fig. 1c). To handle such complex distribution of *H*_*S*_, we developed a Hurdle-beta Bayesian model [88] to separate the zero structure (absence of genetic diversity) from the non-zero structure (presence of genetic diversity) of data. The Hurdle model is defined as a finite mixture of two processes: a degenerate distribution with point mass and zero, which determines the presence or absence of data—in our case *H*_*S*_ values above or below the value of 0.009, respectively— and a conditional-to-presence continuous process supported by the open interval between the lowest non-zero *H*_*S*_ value and 1. The first process (absence of genetic diversity) was modeled with a Bernoulli distribution, whilst the continuous component (presence of genetic diversity) was modeled with a Beta distribution (see the full development of the model in the Supporting Information section). The predictors of the model were the 19 bioclimatic variables available from WorldClim and topsoil pH available from The Geographical Database from Eurasia. The Watanabe–Akaike information criterion (WAIC) [89]; was computed to determine the best models. The model included a stochastic spatial effect (the spatial term) to remove the spatial autocorrelation of data [56].

### Drivers of genetic differentiation

We estimated the ecological, genetic and evolutionary drivers of genetic differentiation of Iberian *A. thaliana* as follows. IBD was based on geographic distance. However, instead of using the Euclidian distance among population pairs, we created a raster by assigning a 0.5 value to all 1-km^2^ pixels [90, 91] of the Iberian Peninsula map. We then calculated pairwise distances, known as geographic resistance distance, among all *A. thaliana* populations employing the new raster. We did not use the Euclidean distance to compute IBD because straight lines between population pairs may introduce some bias when such straight lines traverses natural barriers for the study organism (i.e. the coastline or open sea) [92].

IBE was based on the 19 bioclimatic variables. In addition, we used The CORINE Land Cover to obtain the percentage of human-modified habitat (i.e. urban areas, crops and semi-natural grasslands) within a 500 m radius from the population GPS coordinate [39]. The percentages of human-modified and natural habitat were significantly negatively correlated (*N* = 278; *r* = − 0.95; *P* < 0.001; Pearson’s correlation) and nearly summed to 100%. Finally, a topsoil pH layer was also used. Combining these 21 environmental variables, we conducted a principal component analysis (PCA) with varimax rotation using the software SPSS v.25 (IBM, Chicago, IL USA). Thus, we obtained the PC scores of the first three principal components (PC) for each population. We then used the PC scores to calculate environmental dissimilarity between populations as Euclidean distances derived with the ‘dist’ function in R. Furthermore, we also calculated dissimilarity matrices among populations for each PC axis separately, as they were interpretable in terms of environmental gradients.

To compute IBR, we first estimated habitat suitability for *A. thaliana* across the Iberian Peninsula with the maximum-entropy modeling technique implemented in the software Maxent 3.3.3 k [93, 94]. To do that, we used 478 *A. thaliana* populations available to date (collected between 2000 and 2019; Fig. S1), and the same environmental variables used in a previous habitat suitability model [39]. In particular, the model included topsoil pH and eight bioclimatic variables, which were annual mean temperature (BIO1), mean diurnal temperature range (BIO2), isothermality (BIO3), temperature seasonality (BIO4), mean temperature of wettest quarter (BIO8), annual precipitation (BIO12), precipitation seasonality (BIO15) and precipitation of the warmest quarter (BIO18). We applied Maxent using default parameters except for features using the hinge type, making it comparable to a Generalized Additive Model [94]. We obtained consistent results with previous habitat suitability models conducted for Iberian *A. thaliana* with lower sample sizes [39, 56].

Next, we transformed the resulting habitat suitability into resistance values as 1 minus the value of each pixel. Thus, greater pixel values represented lower occurrence probability of *A. thaliana* (higher resistance) and vice-versa. Using circuit theory [24, 25], we examined whether genetic differentiation among *A. thaliana* populations was accounted for by IBR. We used Circuitscape v.4.0 [95] to calculate resistance distance matrices assigning pixel values as resistance values. We calculated resistance distance among all pairs of populations across Iberian Peninsula, as well as among all pairs of populations within each genetic cluster.

To quantify the relative contributions of IBD, IBE and IBR on the genetic differentiation (pairwise *F*_*ST*_ values) among *A. thaliana* populations, we fitted maximum-likelihood population effect models (MLPE) [96]. Code implementing the MLPE correlation structure within the R package nlme v. 3.1–143 comes from the corMLPE package. The model used penalized least squares and a residual covariance structure designed to account for the non-independence of pairwise distances. Given the inherent spatial dependence structure in pairwise comparisons between *A. thaliana* populations, all MLPE considered spatial locations. To this end, we used a modification of the MLPE model incorporating the correlation between pairwise measurements due to comparison of populations and spatial locations (nested MLPE or NMLPE) [91]. Since the independent variables (IBD, IBE and IBR) used in NMLPE had very different units, we normalized them by subtracting the mean and dividing by the standard deviation in all analyses. We did not linearize pairwise *F*_*ST*_ values. We conducted NMLPE on the complete dataset (*N* = 278 populations) as well as on the subset of those populations exhibiting *H*_*S*_ different from zero (*N* = 212 populations with *H*_*S*_ > 0.009). The results were consistent between the two datasets, showing that the 66 populations with no genetic diversity were not biasing the results.

In addition, we ran NMLPE for each genetic cluster to evaluate whether the effects of IBD, IBE and IBR on genetic differentiation differed among genetic clusters. This is because the number of genetic clusters represents the outcome of the demographic and evolutionary history of *A. thaliana* in the region. We considered each population to belong to a specific genetic cluster when the average value of the membership proportions of its individuals was ≥0.3 for that specific cluster. Given the marked genetic structure of the study system, threshold values of 0.25 and 0.40 were consistent, but 0.3 provided a clearer classification of individuals into genetic clusters. The majority of populations were assigned to a single cluster (*N* = 230). We assigned populations reaching the minimum average membership proportion for more than one cluster to multiple clusters. Overall, 47 populations were assigned to two clusters, whereas only one population had average membership proportions higher than 0.3 for three clusters.

We also ran NMLPE on the complete dataset and each cluster separately, but replacing IBE by the PC axes. Thus, we used IBD, PC1, PC2, PC3 and IBR as independent variables. We used the Akaike information criterion (AIC) to compare models containing all possible combinations of non-collinear predictions (*r* < 0.6), created with the ‘dredge’ function from the R package MuMIn v1.4 [97]. In those cases where several models were top-ranked with ∆AIC < 2, we calculated the model averaged parameter estimates (*β*) with standard errors for the explanatory variables included in all top-ranked models also using MuMIn. We considered effect sizes as significant when 95% confidence intervals did not overlap zero.

## Data Availability

Data deposited in the Dryad repository: 10.5061/dryad.47d7wm393 [98]. The tool to retrieve and examine aerial orthophotographs deposited in the Zenodo repository: https://zenodo.org/record/2552031.

## Abbreviations

AIC: Akaike information criterion
BIC: Bayesian information criterion
DAPC: Discriminant analysis of principal components
IBD: Isolation-by-distance
IBE: Isolation-by-environment
IBR: Isolation-by-resistance
MCMC: Monte Carlo Markov chain
NMLPE: Nested maximum-likelihood population effect models
SNP: Single nucleotide polymorphism
WAIC: Watanabe–Akaike information criterion
